# Effect of feeding pineapple waste on growth performance, texture quality and flesh colour of nile tilapia (*Oreochromis niloticus*) fingerlings

**DOI:** 10.1016/j.sjbs.2021.12.027

**Published:** 2021-12-16

**Authors:** Suniza Anis Mohamad Sukri, Yusrina Andu, Zuharlida Tuan Harith, Shazani Sarijan, Mohd Naim Firdaus Pauzi, Lee Seong Wei, Mahmoud A.O. Dawood, Zulhisyam Abdul Kari

**Affiliations:** a Department of Agricultural Sciences, Faculty of Agro-Based Industry, Universiti Malaysia Kelantan, Jeli Campus, 17600 Jeli, Kelantan, Malaysia; b Faculty of Computer and Mathematical Sciences, Universiti Teknologi MARA Negeri Sembilan, Kuala Pilah Campus, 72000 Kuala Pilah, Negeri Sembilan, Malaysia; c Department of Environment and Water Engineering, Faculty of Engineering, Universiti Teknologi Malaysia, 81310 Johor Bahru, Johor, Malaysia; d Department of Agrotechnology and Bio-Industry, Politeknik Jeli, 17600 Jeli, Kelantan, Malaysia; e Department of Animal Production, Faculty of Agriculture, Kafrelsheikh University, 33516 Kafrelsheikh, Egypt; f The Center for Applied Research on the Environment and Sustainability, The American University in Cairo, 11835 Cairo, Egypt

**Keywords:** Feed formulation, Growth performance, Nile tilapia, Pineapple waste, Texture quality

## Abstract

The study aims to evaluate the effects of pineapples waste on the growth, texture quality and flesh colour of Nile tilapia (*Oreochromis niloticus*) fingerlings. Fingerlings were fed with four different levels of pineapple waste diets throughout 56 days, which contain a control group (Diet 1) and experimental diets that formulated with 10% (Diet 2), 20% (Diet 3) and 30% (Diet 4) of pineapple waste. The experimental diet was formulated with rice bran, fish meal, soybean meal, vitamin and mineral premix, vegetable oil and binder to attain 32% dietary protein. The results revealed that the formulated fish diet with pineapple waste given the optimum weight gain, weight gain percentage, specific growth rate than the control group, where Diet 4 has shown the highest value (p < 0.05). There were no effects of the pineapple waste diet on the texture quality of the fillet, while only red chromaticity (a*) showed a significant difference (p < 0.05). In conclusion, the addition of pineapple waste can improve the growth rate of Nile tilapia, and the supplementation level of the pineapple waste in the diet was 30% of the total feed formulation.

## 1 Introduction

Nile tilapia (*Oreochromis niloticus*) is wide culture freshwater species in over 100 countries (Makori et al. 2017). Tilapia is easy to breed, disease-resistant and highly adaptable to a wide range of environmental conditions. To suit local protein needs, tilapia was introduced to undeveloped countries and cultivated on a subsistence level. Tilapia has made its way into mainstream seafood markets as production processes have improved and off-flavors have been reduced. Meanwhile, in highly industrialized countries, only modest markets for live and frozen tilapia were formed and dominated by immigrant groups. In Malaysia, *O. niloticus* is the major fish species for freshwater aquaculture where contributes approximately 90% of the total production of tilapia species. Its production skyrocketed from 28,401 (2005) to 38,642 tones (2010), exhibiting a 36% increment with almost USD60 millions of trades (Azhar, 2014).

On average, aquaculture feeds expenses represent 75 to 95% of the total operating costs in intensive farming systems (El-Sayid et al., 2015). Although the operating costs vary, farmers usually look for new practical alternative feed sources that enhance fish growth and concurrently reduce feed costs (Kari et al., 2022). It is worth noting that the growth performance is affected by the quality of feed and its nutrient content. Since the cost of food items to prepare animal feed is pricy, hence artificial feed is also considered. In general, artificial feed is nutritionally balanced, cost-effective, and growth-efficient feeds. Indeed, suitable ingredients in feed formulation could give the optimum development and survival (Pati et al., 2016). Most countries have the main problem with agricultural waste (i.e: pulp, crop residues, leaf litter, fruit, and vegetable by-products) to fulfil the food supply worldwide. To eliminate or recycling such waste is costly hence, a swift resolution and strategies are pertinent to prevent environmental pollution. Several studies have documented the use of various waste materials in the composition of aquaculture feed. The substitution of various waste products in aquaculture feed formulation was reported in several studies. For instance, as reported by Tonsy et al. (2019), the utilization of orange peel waste has given a significant effect to the growth performance of *O. niloticus*. Meanwhile, apple peel waste has been reported as a good potential feed additive for genetically improved farmed tilapia (GIFT, *O. niloticus*) (Qiang et al. 2019). In another study by Abdul Kari et al., 2021, partial replacing with the fish meal with fermented soy pulp can enhance the growth performance and healthier African catfish, *Clarias gariepinus*. It shows that agricultural waste from agro-based industry may be useful to animal feeding with a comprehensive study implementation.

Malaysia is one of the biggest pineapple producers worldwide besides Thailand, the Philippines, Indonesia, China, Kenya, and India (Lasekan and Hussein, 2018). Currently, there are approximately 16,500 ha of pineapple plantations in Malaysia. It is worthy of note that about 59.36% of pineapples are considered agro-industrial waste (Sukruansuwan and Napathorn, 2018). Pineapple waste is used as a fertiliser and animal feed occasionally. However, the trash is frequently burned or dumped on the ground, where it is susceptible to microbial deterioration and poses an environmental threat. Pineapple waste contains has a high sugars, carbohydrates, protein, and fibre contents (Ketnawa et al. 2012). It also contains bromelain, bioactive and functional compounds and is considered a dietary supplement (Azizan et al., 2020, Pavan et al., 2012). It has acceptable levels of antinutrients factor such as tannin and phytic acid (Bakri et al., 2020). In addition, the waste can be exploited as a high-quality animal feed through the Solid-State Fermentation (SSF) technique to increase their protein contents (Aruna et al., 2019). Thus, it reveals that pineapple waste has a significant advantage to be converted as value-added products.

Most of the findings shown agricultural waste has no negative consequences effects on the studied fish. Notwithstanding, the most important characteristics are growth performance and feeding efficiency before considering the substitute by-product as an alternative source of aquaculture feed. The main concern is whether the usage of agricultural waste in feed can affect the seafood texture and colour. Indeed, the texture and colour of fish flesh are crucial in the aquaculture industry to sustain quality control and product development. Therefore, as a result, the goal of this study is to see how pineapple waste affects the growth of *O. niloticus*. Texture quality analysis and colour evaluation of the flesh were also carried out since both contribute to consumer acceptance and marketability.

## 2 Materials and methods

### 2.1 Ethics statements

This study was carried out at laboratory animals and fish hatchery and was approved by the Animal Ethical Committee of Universiti Malaysia Kelantan (Malaysia)

### 2.2 Experimental diets and design

Pineapples were obtained from a private farm in Jeli, Kelantan, Malaysia. The pineapple waste, including leaves, crowns and peel, was sliced and chopped into smaller pieces and dried for 48 h at 70 °C. Then, the dry waste was then crushed in a heavy-duty blender and filtered through a screen with a mesh size of 250 µm to produce a fine powder. Four experimental diets were prepared based on common requirements of fish nutrition. Table 1 shows the different levels of diets where Diet 1 (0% of pineapple waste), Diet 2 (10% of pineapple waste), Diet 3 (20% of pineapple waste) and Diet 4 (30% of pineapple waste). To attain 32% of dietary protein, the specified feedstuff and pineapple wastes were mixed together. Feed formulation was carried out using Winfeed Software (Winfeed 2.8), and the chemical analysis and formulation content of the trial diets are shown in Table 1. Chemical analysis and diets were conducted according to AOAC method (2012).

**Table 1:** Chemical analysis and fish feed formulation diet.

Ingredients	Diet 1 (g)	Diet 2 (g)	Diet 3 (g)	Diet 4 (g)
Rice bran	20.75	18.25	15.75	13.25
Fish meal	20.75	18.25	15.75	13.25
Corn meal	20.75	18.25	15.75	13.25
Soybean meal	20.75	18.25	15.75	13.25
Vitamin and mineral premix^2^	2	2	2	2
Vegetable oil^1^	5	5	5	5
Binder	10	10	10	10
Pineapple waste	0	10	20	30
Total	100	100	100	100
Proximate analysis (%)				
Crude protein	32	32	32	32
Crude Fat	4.53	4.26	4.43	4.33
Crude fiber	6.54	6.88	7.23	7.33
Moisture	8.18	8.03	7.80	7.94
Ash	10.60	9.53	9.55	9.43
Energy	3200	3200	3200	3200

*^1^Visawit vegetable oil *^2^ Vitamin and Mineral premix (g/kg pemix): Vitamin C, KCL, 90; KI, 0.04; CaHPO_4_·2H_2_O, 500; NaCl, 40; CuSO_4_·5H_2_O, 3; ZnSO_4_·7H_2_O, 4; CoO_4_, 0.02; FeSO_4_.7H_2_0, 20; MnSO_4_·H_2_O, 3: CaCo_3_, 215; MgOH, 124: Na_2_SeO_3_, 0.03; NaF1

### 2.3 Fish and culture condition

The healthy farm-raised fingerlings were obtained from the local hatchery in Jeli, Kelantan. All fingerlings were acclimatized for seven days to prevent stress and fed commercial pellets (TP0, Star Feedmills, Thailand contain 32% crude protein, 3% crude fat) before diet treatments. At the beginning of the experiment, triplicate groups of 30 healthy fingerlings with an approximate weight of 6.0 ± 1.0 g were arbitrarily selected and reared in 12 aquaria 25L. All experimental fingerlings were fed three times daily for eight weeks. Through the experiment, water quality measures such as temperature, pH, dissolved oxygen, ammonia level, total alkalinity and salinity were recorded. To maintain water quality, detritus, uneaten feed and dead fish were syphoned out of the tank, and half of the water was swapped daily (Soundarapandian et al., 2009, Sukri et al., 2016).

### 2.4 Growth performance

At the end of feeding trial session, each fingerling was individually weighed. The following formulas were used to compute the Weight Gain (WG), Specific Growth Rate (SGR), Percentage of Survival and Feed Conversion Ratio (FCR):

$$Weight\;Gain\;\left(WG\right)=W_{f}-W_{i}$$

$$Specific\;Growth\;Rate\;\left(SGR\right)=\frac{\left(logW_{f}-logW_{i}\right)}{T}\times 100$$

where,

$$W_{f}=Fin\mathrm{al}\;fish\;weight\;\left(g\right),$$

$$W_{i}=Initial\;fish\;weight\;\left(g\right)$$

T = experimental period in days.

$$Survival\;Rate\;\left(SR\%\right)=\frac{N_{f}}{N_{i}}x100$$

where,

$$N_{f}=the\;number\;of\;fish\;stock\;at\;the\;end\;\mathrm{of}\;experiment,$$

$$N_{i}=the\;numb\mathrm{er}\;of\;fish\;stock\;at\;the\;beginning\;of\;the\;experiment.$$

$$Feed\;Conversion\;Ration\;(FCR)=\frac{Total\;weight\;of\;dry\;feed\;given}{Total\;wet\;weight\;gain}$$

### 2.5 Texture analysis

Texture profile studies of tilapia were performed after eight weeks of the trial, including hardness, chewiness, springiness, cohesiveness, and gumminess using Brookfield CT3 Texture Analyzer. Three fillet samples of each replicate of treatments were evaluated for texture quality. The fillet was cut from head to tail and dorsal to ventral according to Bland et al. (2018) method. Fillet samples were placed on the stage of texture analyser and compressed with a cylinder probe. Samples were compressed twice as has been prescribed in the protocol: a pre-test speed, a test speed, target value and trigger value were set as 1.0 mm/s, 10.00 mm/s, 1.00 cm and 5 g, respectively.

### 2.6 Colour evaluation

The flesh colour was evaluated according to Cheng et al. (2015) method where L* is indicated as lightness, a* is redness and b* is yellowness using CR-400 Chroma meter (Konica Minolta, Osaka, Japan).

### 2.7 Statistical analysis

One-way variance analysis (ANOVA) and Duncan’s Multiple Range test were used to analyse the data. The statistical analysis was performed using SPSS Version 25 at 0.05 significant level (Hazra et al., 2020).

## 3 Results

### 3.1 Effects of pineapple waste on the growth performance

Table 2 shows the growth performance of Nile tilapia. Diet 1 showed the highest survival rate throughout the study. Meanwhile, in Table 3, Diet 4 recorded the lowest FCR among all treatments. The results, however, show that there was no statistically significant difference in both parameters (p > 0.05). Contrariwise, weight gain, percentage of weight gain, and specific growth rate were significantly higher in the pineapple waste diet-fed fish than control treatment (p < 0.05). Thus, the present study indicated the addition of pineapple waste, even at a different level, have increased the weight gain of Nile tilapia fingerlings. Of these, Diet 4 gave the best performance as the weight gain, percentage of weight gain, and specific growth rate were significantly superior compared to other diets (p < 0.05).

**Table 2:** Growth performance of Nile tilapia with average mean ± SD.

Treatment	Initial Weight (g)	Final Weight (g)	Weight Gain (g)	Percentage of Weight Gain (%)	Specific Growth Rate (%)
Diet 1	6.67 ± 0.19^a^	14.23 ± 0.31^a^	7.57 ± 0.12^a^	113.54 ± 1.59^a^	0.59 ± 0.01^a^
Diet 2	6.83 ± 0.24^a^	14.87 ± 0.41^a^	8.03 ± 0.12^ab^	117.66 ± 2.98^a^	0.60 ± 0.01^a^
Diet 3	6.73 ± 0.25^a^	15.13 ± 0.25^a^	8.40 ± 0^b^	124.93 ± 4.69^a^	0.63 ± 0.02^a^
Diet 4	6.80 ± 0.28^a^	16.47 ± 0.68^b^	9.67 ± 0.52^c^	142.30 ± 7.71^b^	0.69 ± 0.03^b^

Diet 1 = contain 0% of pineapple waste, Diet 2 = contain 10% of pineapple waste, Diet 3 = contain 20% of pineapple waste, Diet 4 = contain 30% of pineapple waste.

**Table 3:** Feed Conversion ratio (FCR) and survival rate of Nile tilapia with mean ± SD.

Treatment	Feed Conversion Ratio	Survival Rate (%)
Diet 1	4.91 ± 0.62^a^	71.11 ± 15.48^a^
Diet 2	4.32 ± 0.25^a^	64.45 ± 3.14^a^
Diet 3	4.03 ± 0.46^a^	61.11 ± 11.33^a^
Diet 4	3.87 ± 0.49^a^	65.56 ± 8.31^a^

Diet 1 = contain 0% of pineapple waste, Diet 2 = contain 10% of pineapple waste, Diet 3 = contain 20% of pineapple waste, Diet 4 = contain 30% of pineapple waste.

### 3.2 Texture analysis

Table 4 shows the hardness, cohesiveness, springiness, gumminess and chewiness of tilapia fed different levels of pineapple waste. Although there was no significant difference among treatments, the study found that fish diet with pineapple waste (Diet 2 to 4) showed a high hardness value than control (Diet 1). Of these, Diet 4 recorded the highest value. The study also showed that the means value of chewiness for Diet 2 (37.00 mJ) and Diet 1 (0.40 mJ) was the highest compared to Diet 3 (-0.32 mJ) and Diet 4 (-1.23 mJ). The means value of cohesiveness was decreased from 7.25 for Diet 1 to −0.40 for Diet 3. As for springiness, Diet 1 shows the highest value at 1.02 cm. The gumminess for Diets 2 and 4 showed the highest value at 1001.00 ± 915.03 g and 3474.11 ± 2817.56 g than of the control diet at only 763.45 ± 125.51 g.

**Table 4:** Hardness, cohesiveness, springiness, gumminess and chewiness values of each sample in different experimental diets.

Treatment/Parameter	Hardness (g)	Cohesiveness	Springiness (cm)	Gumminess (g)	Chewiness (mJ)
Diet 1	80.11 ± 3.97^a^	7.25 ± 2.00^a^	1.02 ± 0.54^a^	763.45 ± 125.51^a^	0.40 ± 31.64^a^
Diet 2	107.78 ± 46.00^a^	0.03 ± 5.05^a^	0.29 ± 0.31^a^	1001.00 ± 915.03^a^	37.00 ± 24.19^a^
Diet 3	81.17 ± 8.26^a^	−0.40 ± 5.48^a^	−0.04 ± 0.00^a^	337.67 ± 39.37^a^	−0.32 ± 0.13^a^
Diet 4	149.89 ± 7.86^a^	2.74 ± 0.77^a^	−0.02 ± 0.00^a^	3474.11 ± 2817.56^a^	−1.27 ± 0.33^a^

^1^Value are mean ± SE. Data within the same column with different superscript letters are significantly (p < 0.05).

^2^Diet 1 = contain 0% of pineapple waste, Diet 2 = contain 10% of pineapple waste, Diet 3 = contain 20% of pineapple waste, Diet 4 = contain 30% of pineapple waste.

### 3.3 Colour evaluation

The present results in Table 5 showed that although the lightness chromaticity (L*) was not statistically significant, Diet 2 had a higher value than Diet 1 (without pineapple waste). Meanwhile, Diet 2 to 4 showed the lowest value of yellow chromaticity (b*) compared with Diet 1 (p > 0.05). Only red chromaticity (a*) shows a significant difference in all treatments (p < 0.05) as the value of redness of Diet 4 was higher than Diet 1.

**Table 5:** Lightness (L*), red chromaticity (a*) and yellow chromaticity (b*) values of each sample in different experimental diets.

Treatment/Parameters	L*	a*	b*
Diet 1	34.08 ± 0.41^a^	9.01 ± 0.18^b^	9.31 ± 0.17^a^
Diet 2	34.38 ± 1.05^a^	7.96 ± 0.24^c^	8.82 ± 0.25^ab^
Diet 3	33.34 ± 0.78^a^	8.55 ± 0.43^bc^	8.96 ± 0.16^ab^
Diet 4	33.49 ± 0.53^a^	9.91 ± 0.08^a^	8.29 ± 0.34^b^

^1^Value are mean ± SE. Data within the same column with different superscript letters are significantly (p < 0.05).

^2^Diet 1 = contain 0% of pineapple waste, Diet 2 = contain 10% of pineapple waste, Diet 3 = contain 20% of pineapple waste, Diet 4 = contain 30% of pineapple waste.

## 4 Discussion

Our present findings were similar to the recent studies by Van Doan et al., 2021, Yuangsoi et al., 2018, which reported the optimum growth performance of Nile tilapia. Meanwhile, Deka et al. (2003) have previously reported that the best growth performance of rohu (*Labeo rohita*) fingerlings was by feeding a diet with 25% pineapple waste. Their study also found that other fruits processing waste such as orange and sweet lime at a level of 25% are saved for rohu fingerlings to consume in the diet. Indeed, the agricultural waste addition in animal feed can reduce the environmental issue and simultaneously increase farmers' economy.

The addition of supplements in fish feed is a common practice to enhance the optimum growth and healthy fish cultivated on the farm. For instance, Zulhisyam et al. (2021) reported that the substitution of 50% fish meals with fermented soy pulp showed better growth performance of *C. gariepinus*. Besides, the livestock industry has also utilised agriculture waste in animal feeding to increase feed conversion. Pineapple skin (peels) contains a high concentration of bromelain followed by core, crown and stem (Misran et al. 2019). Bromelain is an enzyme that helps the texture of flesh become more tender and soft. It also reduces inflammation in the body. The active ingredients in bromelain are the mixture of cysteine proteases. These enzymes break down the proteins in the food, releasing small peptides. It is critical to improve growth performance by increasing protein digestibility and rapid absorption. A previous study has shown that the supplementation of exogenous protease treatment improved the growth performance of gibel carp, *Carassius auratus gibelio* (Shi et al. 2016). Their study also reported the apparent digestibility of dry matter, crude protein, protein and lipid retention. Meanwhile, according to Li et al. (2016) adding of protease in a low fish meal diet can improve white shrimp, *Litopenaeus vannamei* and tilapia, *Oreochromis niloticus × O. aureus* growth, where the growth performance was similar to those fed a high fish meal diet.

The present results are also parallel to other animals which fed pineapple waste. For instance, broiler fed pineapple leaf powder has significantly increased the weight gain, growth performance and feed conversion ratio (Rahman and Yang, 2018). Similar results were also observed for ruminants fed pineapple waste (Adekanbi et al., 2017, Costa et al., 2007). It shows that the flavour and smell of pineapple waste are acceptable to be consumed by livestock. Hence, the addition of pineapple waste into fish and livestock feeds do not produce any adverse effects. The pineapple waste also has bioactive compounds that can alter the gut microbiome to enhance digestion and assimilation of nutrients (Rahman and Yang, 2018). In addition, the high fibre content in the pineapple waste plays a significant role in reducing digestion of other dietary components that affect the efficiency of digestibility and absorption of nutrients.

Good aquatic diets are crucial for fish health, texture, and colouration. It also improved the water quality and reproductive potential of farmed fish. The characteristics of raw meat represent the overall quality and acceptability of the fish product. There are no standard parameters to determine the physical meat quality of most edible species (Komolka et al. 2020). However, texture property assessment could exhibit the quality of fish and fish-based products (Gonçalves et al., 2018, Hultmann and Rustad, 2002, Klinc et al., 2009). Certainly, freshness is the primary aspect in assessing the quality of fish meat as it is directly related to the consumers' perception of the appearance, texture, and taste.

Jun et al. (2014) reported that the dystrophin disappeared quickly after the fish died. Hence this resulted in the detachment of myofibers and myocommata and a reduction in textural resilience. Meanwhile, the actin and desmin collapsed until the muscle tissues exhibited a decayed sensory appearance and texture. Bromelain, which is derived from pineapple peels, has previously been shown to successfully tenderise beef, poultry and squid (Ketnawa and Rawdkuen, 2011). Bromelain is an inexpensive enzyme and has become an effective tendering alternative for meat and has attracted considerable attention from the industry (Arshad et al. 2014). The flesh texture regularly depends on the fish type or species, their size and age, fat content and distribution and muscle density (Hultman and Rustad, 2002). The flesh texture of fish can also be affected by the muscle metabolism post-mortem mechanisms. These mechanisms include the rigour mortis process, proteolysis, microbiological process and storage environments (Hultman and Rustad, 2002).

Chewiness is the energy needed to chew a solid sample into a steady state of swallowing (Zhao et al., 2017). The decrease in chewiness value in the present study indicates the breakdown of peptide and disulphide bonds in myofibrillar proteins structure by bromelain (Feng et al., 2017, Shin et al., 2008). Cohesiveness refers to a material’s ability to accept a second deformation in comparison to its resistance to the first (Floury et al. 2009). It shows that the diets that contain pineapple waste have a substantial effect on gumminess. The present study postulates that the bromelain enzyme may probably affect the flesh texture of the Nile tilapia. Thus, our result corresponds with previous studies demonstrating the tenderness of meat increased after applying bromelain (Calkins and Sullivan, 2007).

The colour of the flesh is crucial to determine the consistency of the fish fillets, such as white (lightness), red, and yellow. In general, white fillets are of the best quality, while pink and red represent poor bleeding techniques (Sørensen, 2005). However, the colour of the flesh is also related to the fish species. Ponsano et al. (2014) reported that besides white fish, the red colour of some fish species is also preferred of which create a trend in the market. Thus, other than freshness, colour qualities are also one of the main criteria in consumers' perception before purchasing meat products. It indicates that consumers' perception is affected by their level of knowledge and sensory appeal (Thongdonphum et al. 2016).

According to Sørensen (2005), the yellow fillet represents the low colour quality caused by the poor water quality in fish farming. Our study cultivates the fish in the glass aquarium, where the water quality is monitored within an optimum range. Hence, we postulate that the low value of yellow chromaticity is probably affected by the compounds in the pineapple waste. Shekarabi et al. (2020) also reported the significant increment of redness of the rainbow trout (*Oncorhynchus mykiss*) fillet fed a diet with black mulberry (*Morus nigra*) juice powder. Their study also found that the carotenoid concentrations in mulberry influence the flesh colourimeter as its high contents increased the redness and yellowness while reducing the lightness of the flesh (p < 0.05). Ponsano et al. (2014) reveal that the carotenoids fed to Nile tilapia increased the red (p < 0.05) and yellow (p > 0.05) values of the fish flesh. Hence, the present finding corresponds with previous studies except for the yellow chromaticity (Brown and Shahidi, 1997, Rahman et al., 2016).

Unlike farmed fish, the flesh colour of wild fish in the natural ecosystem reaches more redness as they consume krill and phytoplankton (Breithaupt, 2007). Most fish on the farm have pale and greyish skin and fillet compared to those obtained in nature (Diler and Dilek, 2002). As to improve this, previous studies have utilised synthetic or natural sources of carotenoid in fish feeding. Hardy and Lee (2010) reveal that the salmonids skin and muscle colour improved after consumed carotenoids pigments such as astaxanthin in the fish feed. We postulate that the feed formulation with pineapple waste used in the present study has the same ability as astaxanthin to improve the redness colour of the tilapia flesh, particularly for farmed fish. It is also suggested that the redness colour of tilapia flesh could garner customers perception towards the improvement of flesh quality by noting the high content of carotenoids.

## 5 Conclusion

The present study showed the feed formulation with pineapple waste could improve the growth performance of Nile tilapia. The supplementation level of the pineapple waste in the diet was 30% of the total feed formulation for the Nile tilapia fingerlings. There were no significant effects on the texture quality. Diet supplemented with pineapple waste has shown that only red colour was significant and can improve the redness of fillet in farmed fish.

## Declaration of Competing Interest

The authors declare that they have no known competing financial interests or personal relationships that could have appeared to influence the work reported in this paper.
