# Supplementary material for: Effect of feeding pineapple waste on growth performance, texture quality and flesh colour of nile tilapia (Oreochromis niloticus) fingerlings
Source: Saudi J Biol Sci. 2021 Dec 16;29(4):2514–9. doi: 10.1016/j.sjbs.2021.12.027 (PMC9073014; doi:10.1016/j.sjbs.2021.12.027)
Supplement: Supplementary data 1 [file mmc1.pdf]

Effect of feeding pineapple waste on growth performance, texture quality and flesh colour of nile tilapia (Oreochromis niloticus) fingerlings

by Suniza Suniza

Submission date: 22-Oct-2021 08:51PM (UTC+0800)
Submission ID: 1681020841
File name: turnitin.docx (46.67K)
Word count: 4933
Character count: 28120

1. Introduction

Nile tilapia (*Oreochromis niloticus*) is wide culture freshwater species in over 100 countries (Makori et al. 2017). Tilapia is easy to breed, disease-resistant and highly adaptable to a wide range of environmental conditions. To suit local protein needs, tilapia was introduced to undeveloped countries and cultivated on a subsistence level. Tilapia has made its way into mainstream seafood markets as production processes have improved and off-flavors have been reduced. Meanwhile, in highly industrialized countries, only modest markets for live and frozen tilapia were formed and dominated by immigrant groups (FAO, 2020). In Malaysia, *O. niloticus* is the major fish species for freshwater aquaculture where contributes approximately 90% of the total production of tilapia species. Its production skyrocketed from 28,401 (2005) to 38,642 tones (2010), exhibiting a 36% increment with almost USD60 millions of trades (Azhar, 2014).

Aquaculture feeds expenses represent almost 70% of the total operating costs in intensive farming systems. Although the operating costs vary, farmers usually look for new practical alternative feed sources that enhance fish growth and concurrently reduce feed costs (Zulhisyam et al. 2021). Most countries have the main problem with agricultural waste (i.e: pulp, crop residues, leaf litter, fruit, and vegetable by-products) to fulfil the food supply worldwide. To eliminate or recycling such waste is costly hence, a swift resolution and strategies are pertinent to prevent environmental pollution. Several studies have documented the use of various waste materials in the composition of aquaculture feed. The substitution of various waste products in aquaculture feed formulation was reported in several studies. For instance, as reported by Tonsy et al. (2019), the utilization of orange peel waste has given a significant effect to the growth performance of *O. niloticus*. Meanwhile, apple peel waste has been reported as a good potential feed additive for genetically improved farmed tilapia (GIFT, *O. niloticus*) (Qiang et al. 2019). It shows that agricultural waste from agro-based industry may be useful to animal feeding with a comprehensive study implementation.

Malaysia is one of the biggest pineapple producers worldwide besides Thailand, the Philippines, Indonesia, China, Kenya, and India (Lasekan and Hussein, 2018). Currently, there are approximately 16,500 hectares of pineapple plantations in Malaysia. It is worthy of note that about 59.36% of pineapples are considered agro-industrial waste

(Sukruansuwan and Napathorn, 2018). Pineapple waste is used as a fertiliser and animal feed on accasion. However, the trash is frequently burned or dumped on the groud, where it is susceptible to microbial deterioration and poses an environmental threat.. Pineapple waste contains has a high sugars, carbohydrates, protein, and fibre contents (Ketnawa et al. 2012). It also contains bromelain, bioactive and functional compounds and is considered a dietary supplement (Azizan et al. 2020; Pavan et al. 2012). Thus, it reveals that pineapple waste has a significant advantage to be converted as value-added products.

Most of the findings shown agricultural waste has no negative consequences effects on the studied fish. Notwithstanding, the most important characteristics are growth performance and feeding efficiency before considering the substitute by-product as an alternative source of aquaculture feed. The main concern is whether the usage of agricultural waste in feed can affect the seafood texture and colour. Indeed, the texture and colour of fish flesh are crucial in the aquaculture industry to sustain quality control and product development. Therefore, as a result, the goal of this study is to see how pineapple waste affects the growth of *O. niloticus*. Texture quality analysis and colour evaluation of the flesh were also carried out since both contribute to consumer acceptance and marketability.

7

2. Materials and Methods

2.1 Ethics statements

4

This study was carried out at laboratory animals and fish hatchery and was approved by the Animal Ethical Committee of Universiti Malaysia Kelantan (Malaysia)

2.2 Experimental diets and design

Pineapples were obtained from a private farm in Jeli, Kelantan, Malaysia. The pineapple waste, including leaves, crowns and peel, was sliced and chopped into smaller pieces and dried for 48 hours at 70°C. Then, the dry waste was then crushed in a heavy-duty blender and filtered through a screen with a mesh size of 250 microns to produce a fine powder. Four experimental diets were prepared based on common requirements of fish nutrition. Table 1 shows the different levels of diets where Diet 1 (0% of pineapple

waste), Diet 2 (10% of pineapple waste), Diet 3 (20% of pineapple waste) and Diet 4 (30% of pineapple waste). To attain 32% of dietary protein, the specified feedstuff and pineapple wastes were mixed together. Feed formulation was carried out using Winfeed Software (Winfeed 2.8), and the chemical analysis and formulation content of the trial diets are shown in Table 1. Chemical analysis and diets were conducted according to AOAC method (2012).

Table 1: Chemical analysis and fish feed formulation diet

Ingredients	Diet 1 (g)	Diet 2 (g)	Diet 3 (g)	Diet 4 (g)
Rice bran	20.75	18.25	15.75	13.25
Fish meal	20.75	18.25	15.75	13.25
Corn meal	20.75	18.25	15.75	13.25
Soybean meal	20.75	18.25	15.75	13.25
Vitamin and mineral premix[2]	2	2	2	2
Vegetable oil[1]	5	5	5	5
Binder	10	10	10	10
Pineapple waste	0	10	20	30
Total	100	100	100	100
Proximate analysis (%)				
Crude protein	32	32	32	32
Crude Fat	4.53	4.26	4.43	4.33
Crude fiber	6.54	6.88	7.23	7.33
Moisture	8.18	8.03	7.80	7.94
Ash	10.60	9.53	9.55	9.43
Energy	3200	3200	3200	3200

*[1]Visawit vegetable oil *[2] Vitamin and Mineral premix (g/kg pemix): Vitamin C, KCL, 90; KI, 0.04; $CaHPO_4.2H_2O$, 500; NaCl, 40; $CuSO_4.5H_2O$, 3; $ZnSO_4$. $7H_2O$, 4; CoO_4, 0.02; $FeSO_4.7H_20$, 20; $MnSO_4.H_2O$, 3: $CaCo_3$, 215; MgOH, 124: Na_2SeO_3, 0.03; NaF1

2.3 Fish and culture condition

The healthy farm-raised fingerlings were obtained from the local hatchery in Jeli, Kelantan. All fingerlings were acclimatized for seven days to prevent stress and fed commercial pellets before diet treatments. At the beginning of the experiment, triplicate groups of 30 healthy fingerlings with an approximate weight of 6.0 ± 1.0 g were arbitrarily selected and reared in 12 aquaria. All experimental fingerlings were fed three times daily for eight weeks. Through the experiment, water quality measures such as temperature,

pH, dissolved oxygen, ammonia level, total alkalinity and salinity were recorded. To maintain water quality, detritus, uneaten feed and dead fish were syphoned out of the tank, and half of the water was swapped daily (Soundarapandian et al. 2009; Sukri et al. 2016).

2.4 *Growth performance*

At the end of feeding trial session, each fingerling was individually weighed. The following formulas were used to compute the Weight Gain (WG), Specific Growth Rate (SGR), Percentage of Survival and Feed Conversion Ratio (FCR):

$$\text{Weight Gain (WG)} = W_f - W_i$$

$$\text{Specific Growth Rate (SGR)} = \frac{(\log W_f - \log W_i)}{T} \text{ x } 100$$

where,

W_f = Final fish weight (g),

W_i = Initial fish weight (g),

T = experimental period in days.

$$\text{Survival Rate (SR \%)} = \frac{N_f}{N_i} \text{ x } 100$$

where,

N_f = the number of fish stock at the end of experiment,

N_i = the number of fish stock at the beginning of the experiment.

$$\text{Feed Conversion Ration (FCR)} = \frac{\text{Total weight of dry feed given}}{\text{Total wet weight gain}}$$

2.5 *Texture analysis*

Texture profile studies of tilapia were performed after eight weeks of the trial, including hardness, chewiness, springiness, cohesiveness, and gumminess using Brookfield CT3 Texture Analyzer. Three fillet samples of each replicate of treatments were

evaluated for texture quality. The fillet was cut from head to tail and dorsal to ventral according to Bland et al. (2018) method. Fillet samples were placed on the stage of texture analyser and compressed with a cylinder probe. Samples were compressed twice as has been prescribed in the protocol: a pre-test speed, a test speed, target value and trigger value were set as 1.0 mm/s, 10.00 mm/s, 1.00 cm and 5 g, respectively.

2.6 *Colour evaluation*

The flesh colour was evaluated according to Cheng et al. (2015) method where L* is indicated as lightness, a* is redness and b* is yellowness using CR-400 Chroma meter (Konica Minolta, Osaka, Japan).

2.7 *Statistical analysis*

One-way variance analysis (ANOVA) and Duncan's Multiple Range test were used to analyse the data. The statistical analysis was performed using SPSS Version 25 at 0.05 significant level.

3. Results

3.1 *Effects of pineapple waste on the growth performance*

Table 2 shows the growth performance of Nile tilapia. Diet 1 showed the highest survival rate throughout the study. Meanwhile, in Table 3, Diet 4 recorded the lowest FCR among all treatments. The results, however, show that there was no statistically significant difference in both parameters ($p > 0.05$). Contrariwise, weight gain, percentage of weight gain, and specific growth rate were significantly higher in the pineapple waste diet-fed fish than control treatment ($p < 0.05$). Thus, the present study indicated the addition of pineapple waste, even at a different level, have increased the weight gain of Nile tilapia fingerlings. Of these, Diet 4 gave the best performance as the weight gain, percentage of weight gain, and specific growth rate were significantly superior compared to other diets ($p < 0.05$).

Table 2. Growth performance of Nile tilapia with average mean ± SD

Treatment	Initial Weight (g)	Final Weight (g)	Weight Gain (g)	Percentage of Weight Gain (%)	Specific Growth Rate (%)
Diet 1	6.67 ±0.19[a]	14.23 ±0.31[a]	7.57 ±0.12[a]	113.54 ±1.59[a]	0.59 ±0.01[a]
Diet 2	6.83 ±0.24[a]	14.87 ±0.41[a]	8.03 ±0.12[ab]	117.66 ±2.98[a]	0.60 ±0.01[a]
Diet 3	6.73 ±0.25[a]	15.13 ±0.25[a]	8.40 ±0[b]	124.93 ±4.69[a]	0.63 ±0.02[a]
Diet 4	6.80 ±0.28[a]	16.47 ±0.68[b]	9.67 ±0.52[c]	142.30 ±7.71[b]	0.69 ±0.03[b]

Diet 1= contain 0% of pineapple waste, Diet 2= contain 10% of pineapple waste, Diet 3= contain 20% of pineapple waste, Diet 4= contain 30% of pineapple waste

Table 3. Feed Conversion ratio (FCR) and survival rate of Nile tilapia

Treatment	Feed Conversion Ratio (Mean ± SD)	Survival Rate (%) (Mean ± SD)
Diet 1	4.91 ±0.62[a]	71.11 ±15.48[a]
Diet 2	4.32 ±0.25[a]	64.45 ±3.14[a]
Diet 3	4.03 ±0.46[a]	61.11 ±11.33[a]
Diet 4	3.87 ±0.49[a]	65.56 ±8.31[a]

Diet 1= contain 0% of pineapple waste, Diet 2= contain 10% of pineapple waste, Diet 3= contain 20% of pineapple waste, Diet 4= contain 30% of pineapple waste

3.2 Texture analysis

Table 4 shows the hardness, cohesiveness, springiness, gumminess and chewiness of tilapia fed different levels of pineapple waste. Although there was no significant difference among treatments, the study found that fish diet with pineapple waste (Diet 2 to 4) showed a high hardness value than control (Diet 1). Of these, Diet 4 recorded the highest value. The study also showed that the means value of chewiness for Diet 2 (37.00 mJ) and Diet 1 (0.40 mJ) was the highest compared to Diet 3 (-0.32 mJ) and Diet 4 (-1.23 mJ). The means value of cohesiveness was decreased from 7.25 for Diet 1 to -0.40 for Diet 3. As for springiness, Diet 1 shows the highest value at 1.02 cm. The gumminess for Diets 2 and 4 showed the highest value at 1001.00±915.03 g and 3474.11±2817.56 g than of the control diet at only 763.45±125.51 g.

Table 4. Hardness, cohesiveness, springiness, gumminess and chewiness values of each sample in different experimental diets

Treatment/ Parameter	Hardness (g)	Cohesiveness	Springiness (cm)	Gumminess (g)	Chewiness (mJ)
Diet 1	80.11±3.97^{a}	7.25±2.00^{a}	1.02±0.54^{a}	763.45±125.51^{a}	0.40±31.64^{a}
Diet 2	107.78±46.00^{a}	0.03±5.05^{a}	0.29±0.31^{a}	1001.00±915.03^{a}	37.00±24.19^{a}
Diet 3	81.17±8.26^{a}	-0.40±5.48^{a}	-0.04±0.00^{a}	337.67±39.37^{a}	-0.32±0.13^{a}
Diet 4	149.89±7.86^{a}	2.74±0.77^{a}	-0.02±0.00^{a}	3474.11±2817.56^{a}	-1.27±0.33^{a}

[1]Value are mean±SE. Data within the same column with different superscript letters are significantly ($p<0.05$).
[2]Diet 1= contain 0% of pineapple waste, Diet 2= contain 10% of pineapple waste, Diet 3= contain 20% of pineapple waste, Diet 4= contain 30% of pineapple waste

3.3 Colour evaluation

The present results in Table 5 showed that although the lightness chromaticity (L*) was not statistically significant, Diet 2 had a higher value than Diet 1 (without pineapple waste). Meanwhile, Diet 2 to 4 showed the lowest value of yellow chromaticity (b*) compared with Diet 1 ($p > 0.05$). Only red chromaticity (a*) shows a significant difference in all treatments ($p < 0.05$) as the value of redness of Diet 4 was higher than Diet 1.

Table 5. Lightness (*L**), red chromaticity (*a**) and yellow chromaticity (*b**) values of each sample in different experimental diets

Treatment/Parameters	*L**	*a**	*b**
Diet 1	34.08±0.41^{a}	9.01±0.18^{b}	9.31±0.17^{a}
Diet 2	34.38±1.05^{a}	7.96±0.24^{c}	8.82±0.25^{ab}
Diet 3	33.34±0.78^{a}	8.55±0.43^{bc}	8.96±0.16^{ab}
Diet 4	33.49±0.53^{a}	9.91±0.08^{a}	8.29±0.34^{b}

[1]Value are mean±SE. Data within the same column with different superscript letters are significantly ($p<0.05$).
[2]Diet 1= contain 0% of pineapple waste, Diet 2= contain 10% of pineapple waste, Diet 3= contain 20% of pineapple waste, Diet 4= contain 30% of pineapple waste

4. Discussion

Our present findings were similar to the recent studies by Van Doan et al. (2021) and Yuangsoi et al. (2018), which reported the optimum growth performance of Nile tilapia. Meanwhile, Deka et al. (2003) have previously reported that the best growth performance of rohu (*Labeo rohita*) fingerlings was by feeding a diet with 25% pineapple waste. Their study also found that other fruits processing waste such as orange and sweet lime at a level of 25% are saved for rohu fingerlings to consume in the diet. Indeed, the agricultural waste addition in animal feed can reduce the environmental issue and

simultaneously increase farmers' economy.

The addition of supplements in fish feed is a common practice to enhance the optimum growth and healthy fish cultivated on the farm. Besides, the livestock industry has also utilised agriculture waste in animal feeding to increase feed conversion. Pineapple skin (peels) contains a high concentration of bromelain followed by core, crown and stem (Misran et al. 2019). Bromelain is an enzyme that helps the texture of flesh become more tender and soft. It also reduces inflammation in the body. The active ingredients in bromelain are the mixture of cysteine proteases. These enzymes break down the proteins in the food, releasing small peptides. It is critical to improve growth performance by increasing protein digestibility and rapid absorption. A previous study has shown that the supplementation of exogenous protease treatment improved the growth performance of gibel carp, *Carassius auratus gibelio* (Shi et al. 2016). Their study also reported the apparent digestibility of dry matter, crude protein, protein and lipid retention. Meanwhile, according to Li et al. (2016) adding of protease in a low fish meal diet can improve white shrimp, *Litopenaeus vannamei* and tilapia, *Oreochromis niloticus* × *O. aureus* growth, where the growth performance was similar to those fed a high fish meal diet.

The present results are also parallel to other animals which fed pineapple waste. For instance, broiler fed pineapple leaf powder has significantly increased the weight gain, growth performance and feed conversion ratio (Rahman and Yang, 2018). Similar results were also observed for ruminants fed pineapple waste (Adekanbi et al. 2017; Costa et al. 2007). It shows that the flavour and smell of pineapple waste are acceptable to be consumed by livestock. Hence, the addition of pineapple waste into fish and livestock feeds do not produce any adverse effects. The pineapple waste also has bioactive compounds that can alter the gut microbiome to enhance digestion and assimilation of nutrients (Rahman and Yang, 2018). In addition, the high fibre content in the pineapple waste plays a significant role in reducing digestion of other dietary components that affect the efficiency of digestibility and absorption of nutrients.

Good aquatic diets are crucial for fish health, texture, and colouration. It also improved the water quality and reproductive potential of farmed fish. The characteristics of raw meat represent the overall quality and acceptability of the fish product. There are

no standard parameters to determine the physical meat quality of most edible species (Komolka et al. 2020). However, texture property assessment could exhibit the quality of fish and fish-based products (Gonçalves et al. 2018; Hultman and Rustad, 2002; Kilinc et al. 2009). Certainly, freshness is the primary aspect in assessing the quality of fish meat as it is directly related to the consumers' perception of the appearance, texture, and taste.

Jun et al. (2014) reported that the dystrophin disappeared quickly after the fish died. Hence this resulted in the detachment of myofibers and myocommata and a reduction in textural resilience. Meanwhile, the actin and desmin collapsed until the muscle tissues exhibited a decayed sensory appearance and texture. Bromelain, which is derived from pineapple peels, has previously been shown to successfully tenderise beef, poultry and squid (Ketnawa and Rawdkuen, 2011). Bromelain is an inexpensive enzyme and has become an effective tendering alternative for meat and has attracted considerable attention from the industry (Arshad et al. 2014). The flesh texture regularly depends on the fish type or species, their size and age, fat content and distribution and muscle density (Hultman and Rustad, 2002). The flesh texture of fish can also be affected by the muscle metabolism post-mortem mechanisms. These mechanisms include the rigour mortis process, proteolysis, microbiological process and storage environments (Hultman and Rustad, 2002).

Chewiness is the energy needed to chew a solid sample into a steady state of swallowing (Zhao et al., 2017). The decrease in chewiness value in the present study indicates the breakdown of peptide and disulphide bonds in myofibrillar proteins structure by bromelain (Feng et al. 2017; Shin et al. 2008). Cohesiveness refers to a material's ability to accept a second deformation in comparison to its resistance to the first (Floury et al. 2009). It shows that the diets that contain pineapple waste have a substantial effect on gumminess. The present study postulates that the bromelain enzyme may probably affect the flesh texture of the Nile tilapia. Thus, our result corresponds with previous studies demonstrating the tenderness of meat increased after applying bromelain (Calkins and Sullivan, 2007).

The colour of the flesh is crucial to determine the consistency of the fish fillets, such as white (lightness), red, and yellow. In general, white fillets are of the best quality, while pink and red represent poor bleeding techniques (Sørensen, 2005). However, the

colour of the flesh is also related to the fish species. Ponsano et al. (2014) reported that besides white fish, the red colour of some fish species is also preferred of which create a trend in the market. Thus, other than freshness, colour qualities are also one of the main criteria in consumers' perception before purchasing meat products. It indicates that consumers' perception is affected by their level of knowledge and sensory appeal (Thongdonphum et al. 2016).

According to Sørensen (2005), the yellow fillet represents the low colour quality caused by the poor water quality in fish farming. Our study cultivates the fish in the glass aquarium, where the water quality is monitored within an optimum range. Hence, we postulate that the low value of yellow chromaticity is probably affected by the compounds in the pineapple waste. Shekarabi et al. (2020) also reported the significant increment of redness of the rainbow trout (*Oncorhynchus mykiss*) fillet fed a diet with black mulberry (*Morus nigra*) juice powder. Their study also found that the carotenoid concentrations in mulberry influence the flesh colourimeter as its high contents increased the redness and yellowness while reducing the lightness of the flesh ($p < 0.05$). Ponsano et al. (2014) reveal that the carotenoids fed to Nile tilapia increased the red ($p < 0.05$) and yellow ($p > 0.05$) values of the fish flesh. Hence, the present finding corresponds with previous studies except for the yellow chromaticity (Brown and Shahidi, 1997; Rahman et al., 2016).

Unlike farmed fish, the flesh colour of wild fish in the natural ecosystem reaches more redness as they consume krill and phytoplankton (Breithaupt, 2007). Most fish on the farm have pale and greyish skin and fillet compared to those obtained in nature (Diler and Dilek, 2002). As to improve this, previous studies have utilised synthetic or natural sources of carotenoid in fish feeding. Hardy and Lee (2010) reveal that the salmonids skin and muscle colour improved after consumed carotenoids pigments such as astaxanthin in the fish feed. We postulate that the feed formulation with pineapple waste used in the present study has the same ability as astaxanthin to improve the redness colour of the tilapia flesh, particularly for farmed fish. It is also suggested that the redness colour of tilapia flesh could garner customers perception towards the improvement of flesh quality by noting the high content of carotenoids.

5. Conclusion

The present study showed the feed formulation with pineapple waste could improve the growth performance of Nile tilapia. There were no significant effects on the texture quality. Diet supplemented with pineapple waste has shown that only red colour was significant and can improve the redness of fillet in farmed fish. The supplementation level of the pineapple waste in the diet was 30% of the total feed formulation for the Nile tilapia fingerlings.

Acknowledgements

The author(s) would like to thank Universiti Malaysia Kelantan for funding this research through grant UMKFUND (R/FUND/A0700/00302A/003/2020/00737).

References

Adekanbi, A. O., Onwuka, C. F. I., Oni, A. O., Ojo, V. O. A., Ajayi, F. T., Popoola, M. A., 2017. Performance evaluation and haematological biochemical parameters of West African dwarf goats fed pineapple waste (Ananas comosus) with or without yeast (Saccharomyces cerevisiae) supplementation. Nigeria Journal of Animal Production. 44(3): 342-353. https://doi.org/10.51791/njap.v44i3.598

AOAC – Association of Official Analytical Chemistry. Official Methods of Analysis of the Association of Analytical Chemists International. 20th ed. AOAC, Gaithersburg, MD.

Arshad, Z. I. M., Amid, A., Yusof, F., Jaswir, I., Ahmad, K., Loke, S. P., 2014. Bromelain: an overview of industrial application and purification strategies. Applied Microbiology and Biotechnology. 98: 7283-7297. https://doi.org/10.1007/s00253-014-5889-y

Azizan, A., Lee, A. X., Abdul Hamid, N. A., Maulidiani, M., Mediani, A., Abdul Ghafar, S. Z., Zolkeflee, N. K. Z., Abas, F., 2020. Potentially bioactive metabolites from pineapple waste extracts and their antioxidant and α-glucosidase inhibitory activities by 1H NMR. Foods. 9(2): 173. https://doi.org/10.3390/foods9020173

Bland, J. M., Bett-Garber, K. L., Li, C. H., Brashear, S. S., Lea, J. M., Bechtel, P. J., 2018. Comparison of sensory and instrumental methods for the analysis of texture of cooked individually quick frozen and fresh-frozen catfish fillets. Food Science & Nutrition. 6(6): 1692-1705. https://doi.org/10.1002/fsn3.737

Breithaupt, D. E., 2007. Modern application of xanthophylls in animal feeding e a review, 2007. Trends in Food Science & Technology. 18: 501-506. http://doi.org/10.1016/j.tifs.2007.04.009

Brown, M. J. A., Shahidi, F., 1997. Effects of stocking density on colour characteristics and deposition of carotenoids in cultured Arctic charr (*Salvelinus alpinus*). Food Chem. 59: 107-114. https://doi.org/10.1016/S0308-8146(96)00205-1

Calkins, C. R., & Sullivan, G., 2007. Adding enzymes to improve beef tenderness. Beef facts product enhancement, National cattleman's beef association. Centennial Colorado: Cattlemen's Beef Board.

Costa, R. G., Correia, M. X. C., Da Silva, J. H. V., De Medeiros, A. N., De Carvalho, F. F. R., 2007. Effect of different levels of dehydrated pineapple by-products on intake, digestibility and performance of growing goats. Small Ruminant Research. 71(1-3): 138-143. http://doi.org/10.1016/j.smallrumres.2006.05.012

Deka, A., Sahu, N. P., Jain, K. K., 2003. Utilization of fruit processing wastes in the diet of *Labeo rohita* fingerling. Asian-australasian Journal of Animal Sciences. 16(11): 1661-1665.

Diler, İ., Dilek, K., 2002. Significance of pigmentation and use in aquaculture. Turkish Journal of Fisheries and Aquatic Sciences. 2(1).

Feng, X., Zhu, Y., Liu, Q., Lai, S., Yang, H., 2017. Effects of bromelain tenderisation on myofibrillar proteins, texture and flavour of fish balls prepared from golden pomfret. Food and Bioprocess Technology. 10(10): 1918-1930. https://doi.org/10.1007/s11947-017-1963-7

Floury, J., Camier, B., Rousseau, F., Lopez, C., Tissier, J. P., Famelart, M. H., 2009. Reducing salt level in food: Part 1. Factors affecting the manufacture of model cheese systems and their structure–texture relationships. LWT-Food Science and Technology. 42(10): 1611-1620. https://doi.org/10.1016/j.lwt.2009.05.026

Gonçalves, A. A., Sauza, M. A., Regis, R. C. P., 2018. Effects of different levels of food additives on weight gain, cook-related yield loss, physicochemical and sensorial quality of Nile tilapia fillets (*Oreochromis niloticus*). International Food Research Journal. 25(5): 2068-2080.

Gokoglu, N., Yerlikaya, P., Ucak, I., Yatmaz, H. A., 2017. Effect of bromelain and papain enzymes addition on physicochemical and textural properties of squid (*Loligo vulgaris*). Journal of Food Measurement and Characterization. 11(1): 347-353. https://doi.org/10.1007/s11694-016-9403-3

Hardy, R. W., Lee, C. S., 2010. Aquaculture feed and seafood quality. Bulletin of Fisheries Research and Development Agency. 31: 43-50.

Hultmann, L., Rustad, T., 2002. Textural changes during iced storage of salmon (*Salmo salar*) and cod (*Gadus morbua*). J. Aqua. Food Prod. Tech. 11(3-4): 105-123. https://doi.org/10.1300/J030v11n03_09

Jun-Hue C., D.-W. S., Zhong, H., Xin-An, Z., 2014. Texture and Structure Measurements and Analyses for Evaluation of Fish and Fillet Greshness Quality: A Review. Comprehensive Reviews in Food Science and Food Safety. 13: 52-61. https://doi.org/10.1111/1541-4337.12043

Klinc, B., Cakil, S., Csdun, A., Sen, B., 2009. Effect of phosphate dip treatments on chemical, microbiological, color, textural and sensory changes of rainbow trout (*Oncorynchus mykiss*) fillets during refrigerated storage. Journal of food Product Technology. 18(1-2): 108-119. https://doi.org/10.1080/10498850802581807

Komolka, K., Bochert, R., George, P. F., Yagmur, K., Ralf, P., Bianka, G., 2020. Determination and Comparison of Physical Meat Quality Parameters of Percidae and Salmonidae in Aquaculture. Foods. 9: 388-401. https://doi.org/10.3390/foods9040388

Ketnawa, S., Rawdkuen, S., 2011. Application of BML extract for muscle foods tenderization. Food and Nutrition Sciences. 2: 393–401. https://doi.org/10.4236/fns.2011.25055

Ketnawa, S., Chaiwut, P., Rawdkuen, S., 2012. Pineapple wastes: A potential source for Bromelain Extraction. Food and Bioproducts Processing. 90(3): 385-391. https://doi.org/10.1016/j.fbp.2011.12.006

Lasekan, O., Hussein, F. K., 2018. Classification of different pineapple varieties grown in Malaysia based on volatile fingerprinting and sensory analysis. Chemistry Central Journal. 12(1): 1-12. https://doi.org/10.1186/s13065-018-0505-3

Li, X. Q., Chai, X. Q., Liu, D. Y., Chowdhury, M. A. K., Leng, X. J., 2016. Effects of temperature and feed processing on protease activity and dietary protease on growths of white shrimp, *Litopenaeus vannamei*, and tilapia, *Oreochromis niloticus* × *O. aureus.* Aquaculture Nutrition. 22:1283-1292. https://doi.org/10.1111/anu.12330

Misran, E., Idris, A., Sarip, S. H. M., Ya'akob, H., 2019. Properties of bromelain extract from different parts of the pineapple variety Morris. Biocatalysis and Agricultural Biotechnology. 18: 101095. https://doi.org/10.1016/j.bcab.2019.101095

Pavan, R., Jain, S., Kumar, A., 2012. Properties and therapeutic application of bromelain: a review. Biotechnology Research International. 2012. https://doi.org/10.1155/2012/976203

Ponsano, E. H., Grassi, T. L., Santo, E. F. E. S., Marcos, M. T., Cavazzana, J. F., Pinto, M. F., 2014. Color and carotenoids in tilapia fish fed different carotenoids. In International Congress of Meat Science and Technology. 60. Punta Del Este.

Rahman, M., Yang, D. K., 2018. Effects of *Ananas comosus* leaf powder on broiler performance, haematology, biochemistry, and gut microbial population. Revista Brasileira de Zootecnia. 47. https://doi.org/10.1590/rbz4720170064

Rahman M.M., Khosravi S., Chang K.H., Lee S.M., 2016. Effects of dietary inclusion of astaxanthin on growth, muscle pigmentation and antioxidant capacity of juvenile rainbow trout (On- *corhynchus mykiss*). Prev. Nutr. Food Sci. 2: 281. https://doi.org/10.3746/pnf.2016.21.3.281

Shekarabi, S. P. H., Omidi, A. H., Dawood, M. A., Adel, M., Avazeh, A., Heidari, F., 2020. Effect of black mulberry (*Morus nigra*) powder on growth performance, biochemical parameters, blood carotenoid concentration, and fillet color of rainbow trout. Annals of Animal Science. 20(1), 125-136. https://doi.org/10.2478/aoas-2019-0068

Shi, Z., Li, X. Q., Chowdhury, M. A. K., Chen, J. N., Leng, X. J., 2016. Effects of protease supplementation in low fish meal pelleted and extruded diets on growth, nutrient retention and digestibility of gibel carp, *Carassius auratus gibelio*. Aquaculture. 460: 37-44. https://doi.org/10.1016/j.aquaculture.2016.03.049

Shin, H. G., Choi, Y. M., Kim, H. K., Ryu, Y. C., Lee, S. H., Kim, B. C., 2008. Tenderization and fragmentation of myofibrillar proteins in bovine longissimus dorsi muscle using proteolytic extract from *Sarcodon aspratus*. LWT- Food Science and Technology. 41:1389-1395. https://doi.org/10.1016/j.lwt.2007.08.019

Sørensen, N. K., 2005. Slaughtering processes for farmed Pangasius in Vietnam. Consultancy surveying Pangasius fillet quality and by-products handling in Vietnamese industry. Nofima rapportserie. Fiskeriforskning

Soundarapandian, P., Prakash, K.S., Dinakaran, G.K., 2009. Simple Technology for the Hatchery Seed Production of Giant Palaemonid Prawn *Macrobrachium rosenbergii* (De Man). International Journal of Animal and Veterinary Advances. 1: 49-53.

Sukri, S. A. M., Saad, C. R., Kamarudin, M. S., Yasin, I. S. M., 2016. Effect of different level of *Chlorella* meal on growth and survival of freshwater prawns *Macrobrachium rosenbergii* juvenile. Songklanakarin Journal Science and Technology. 36(6): 641-644

Sukruansuwan, V., Napathorn, S. C., 2018. Use of agro-industrial residue from the canned pineapple industry for polyhydroxybutyrate production by *Cupriavidus necator* strain A-04. Biotechnology for Biofuels. 11(1): 1-15. https:/doi.org/10.1186/s13068-018-1207-8

Thongdonphum, B., Vanichkul, K., Siriwattananon, L., 2016. Effect of dietary protein ingredients from non-toxic agricultural field sources on meat quality of nile tilapia (*Oreochromis niloticus*). International Journal of GEOMATE. 11(28): 2901-2905.

Tonsy, H. D., Ahmed, R. A., Abd-Elghany, F. M., 2019. Effect of orange peel waste on growth performance. Abbassa Int. J. Aqua. 12(1): 131-156. https://doi.org/10.4194/2618-6381-v18_2_06

Van Doan, H., Lumsangkul, C., Hoseinifar, S. H., Tongsiri, S., Chitmanat, C., Musthafa, M. S., El-Haroun, E.,Ringo, E., 2021. Modulation of growth, innate immunity, and disease resistance of Nile tilapia (*Oreochromis niloticus*) culture under biofloc system by supplementing pineapple peel powder and *Lactobacillus plantarum*. Fish & Shellfish Immunology. 155: 212-220. https:/doi.org/10.1016/j.fsi.2021.06.008

Yuangsoi, B., Klahan, R., Charoenwattanasak, S., Lin, S. M., 2018. Effects of supplementation of pineapple waste extract in diet of Nile tilapia (*Oreochromis niloticus*) on growth, feed utilization, and nitrogen excretion. Journal of Applied Aquaculture. 30(3): 227-237. https://doi.org/10.1080/10454438.2018.1439794

Zhao, Y., Yang, X., Li, L., Hao, S., Wei, Y., Cen, J., Lin, H., 2017. Chemical, microbiological, color and textural changes in Nile Tilapia (*Oreochromis niloticus*) fillets sterilized by ozonated water pretreatment during frozen storage. Journal of Food Processing and Preservation. 41(1):e12746. https://doi.org/10.111/jfpp.12746

Zulhisyam, A. K., Kabir, M. A., Dawood, M. A., Razab, M. K. A. A., Ariff, N. S. N. A., Sarkar, T., Pati, S., Edinur, H. A., Mat, K., & Ismail, T. A. (2021). Effect of fish meal substitution with fermented soy pulp on growth performance, digestive enzyme, amino acid profile, and immune-related gene expression of African catfish (*Clarias gariepinus*). Aquaculture, 737418. https://doi.org/10.1016/j.aquaculture.2021.737418

Effect of feeding pineapple waste on growth performance, texture quality and flesh colour of nile tilapia (Oreochromis niloticus) fingerlings

ORIGINALITY REPORT

17%	11%	13%	4%
SIMILARITY INDEX	INTERNET SOURCES	PUBLICATIONS	STUDENT PAPERS

PRIMARY SOURCES

1	Zhao, Yongqiang, Xianqing Yang, Laihao Li, Shuxian Hao, Ya Wei, Jianwei Cen, and Hong Lin. "Chemical, Microbiological, Color and Textural Changes in Nile Tilapia (Oreochromis niloticus) Fillets Sterilized by Ozonated Water Pretreatment During Frozen Storage : Chemical, Microbiological, Color and Textural Changes in Nile Tilapia", Journal of Food Processing and Preservation, 2016.<br>Publication	1%
2	rdo.psu.ac.th<br>Internet Source	1%
3	Submitted to Universiti Putra Malaysia<br>Student Paper	1%
4	Zulhisyam Abdul Kari, Muhammad Anamul Kabir, Mahmoud A.O. Dawood, Mohammad Khairul Azhar Abdul Razab et al. "Effect of fish meal substitution with fermented soy pulp on growth performance, digestive enzyme,	1%

amino acid profile, and immune-related gene expression of African catfish (Clarias gariepinus)", Aquaculture, 2022
Publication

5	onlinelibrary.wiley.com<br>Internet Source	1 %
6	Submitted to Universiti Malaysia Kelantan<br>Student Paper	1 %
7	www.mdpi.com<br>Internet Source	1 %
8	Paul, Sayantan, S. Kulkarni, and K. Jayaraj Rao. "Effect of indian cottage cheese (paneer) - whey on rheological and proofing characteristics of multigrain bread dough", Journal of Texture Studies, 2015.<br>Publication	1 %
9	Dianyu Huan, Xiaoqin Li, Mohiuddin Amirul Kabir Chowdhury, Hang Yang, Gaoyang Liang, Xiangjun Leng. " Organic acid salts, protease and their combination in fish meal - free diets improved growth, nutrient retention and digestibility of tilapia ( ) ", Aquaculture Nutrition, 2018<br>Publication	<1 %
10	espace.curtin.edu.au<br>Internet Source	<1 %

www.tandfonline.com

11	Internet Source	<1 %
12	Hashem H. Abd El-Rahman, Hanan A. Abo-State, Ali S.M. El-Nadi, Hesham Abozaid, Mamdouh I. Mohamed, Abo El-Fetoh M. Abdalla. "Growth Performance, Feed Utilization and Body Composition of Nile Tilapia (Oreochromis niloticus) Fingerlings Fed Moringa (Moringa oleifera Lam.) Seed Meal", Journal of Fisheries and Aquatic Science, 2016<br>Publication	<1 %
13	escipub.com<br>Internet Source	<1 %
14	Submitted to University of Ghana<br>Student Paper	<1 %
15	Submitted to Mansoura University<br>Student Paper	<1 %
16	Rosliana Rosman, Normah Ismail. "Effects of Papaya Leaves Crude Extract on the Physicochemical and Sensory Characteristics of Marinated Chicken Meat", Scientific Research Journal, 2018<br>Publication	<1 %
17	Submitted to Aquinas College<br>Student Paper	<1 %

18 X. Fernandez, M. Bouillier-Oudot, C. Molette, M.D. Bernadet, H. Manse. "Duration of transport and holding in lairage at constant postprandial delay to slaughter—Effects on fatty liver and breast muscle quality in mule ducks", Poultry Science, 2011
Publication
<1 %

19 X.Q. Li, X.Q. Chai, D.Y. Liu, M.A. Kabir Chowdhury, X.J. Leng. " Effects of temperature and feed processing on protease activity and dietary protease on growths of white shrimp, , and tilapia, ", Aquaculture Nutrition, 2016
Publication
<1 %

20 dipot.ulb.ac.be
Internet Source
<1 %

21 sujo-old.usindh.edu.pk
Internet Source
<1 %

22 Elahe Oroji, Mehdi Shamsaie Mehrgan, Houman Rajabi Islami, Issa Sharifpour. "Dietary effect of Ziziphora clinopodioides extract on zootechnical performance, immune response, and disease resistance against Yersinia ruckeri in Oncorhynchus mykiss", Aquaculture Reports, 2021
Publication
<1 %

23 Lumpan Poolsawat, Hang Yang, Yan-Fang Sun, Xiao-Qin Li, Gao-Yang Liang, Xiang-Jun Leng.
<1 %

"Effect of replacing fish meal with enzymatic feather meal on growth and feed utilization of tilapia (Oreochromis niloticus × O. aureus)", Animal Feed Science and Technology, 2021
Publication

24	Submitted to National University of Singapore<br>Student Paper	<1%
25	Xuxia Zhou, Ziqiang Tian, Yanbo Wang, Weifen Li. "Effect of treatment with probiotics as water additives on tilapia (Oreochromis niloticus) growth performance and immune response", Fish Physiology and Biochemistry, 2010<br>Publication	<1%
26	e-sciencecentral.org<br>Internet Source	<1%
27	respiratory-research.biomedcentral.com<br>Internet Source	<1%
28	rif.mak.ac.ug<br>Internet Source	<1%
29	www.entomoljournal.com<br>Internet Source	<1%
30	Sabreen E. Fadl, M.S. ElGohary, Abdelgawad Y. Elsadany, Doaa M. Gad, Farag F. Hanaa, Nagwan M. El-Habashi. "Contribution of microalgae-enriched fodder for the Nile tilapia	<1%

to growth and resistance to infection with Aeromonas hydrophila", Algal Research, 2017
Publication

---

31 X. Li, Y. Wang, Y.Y. Sun, D.D. Pan, J.X. Cao. "The effect of ultrasound treatments on the tenderizing pathway of goose meat during conditioning", Poultry Science, 2018 <1 %
Publication

---

32 Abayomi M. Ajayi, Kayode A. John, Ilerioluwa B. Emmanuel, Emmanuel O. Chidebe, Aduragbenro D.A. Adedapo. "High-fat diet-induced memory impairment and anxiety-like behavior in rats attenuated by peel extract of Ananas comosus fruit via atheroprotective, antioxidant and anti-inflammatory actions", Metabolism Open, 2021 <1 %
Publication

---

33 Amin Avazeh, Milad Adel, Seyed Pezhman Hosseini Shekarabi, Hossein Emamadi et al. " Effects of dietary pomegranate peel meal on the growth performance, blood indices, and innate immune response of rainbow trout ( ) ", Annals of Animal Science, 2020 <1 %
Publication

---

34 Mohamed El‐S. Salem, Heba M. Abdel‐Ghany, Ahmed E. Sallam, Mohamed M. M. El‐Feky, Hebatollah M. Almisherfi. " Effects of dietary orange peel on growth performance, <1 %

antioxidant activity, intestinal microbiota and liver histology of Gilthead sea bream ( ) larvae ", Aquaculture Nutrition, 2019
Publication

35 Nasreen Mohi Alddin Abdulrahman. " Effect of white mulberry (L.) On common carp performance, biological parameters, and blood picture ", Cold Spring Harbor Laboratory, 2021 <1 %
Publication

36 Seyed Pezhman Hosseini Shekarabi, Mehdi Shamsaie Mehrgan, Akbar Banavreh. " Feasibility of superworm, , meal as a partial fishmeal replacer in fingerling rainbow trout, , diet: growth performance, amino acid profile, proteolytic enzymes activity and pigmentation ", Aquaculture Nutrition, 2021 <1 %
Publication

37 hdl.handle.net <1 %
Internet Source

38 link.springer.com <1 %
Internet Source

39 www.cambridge.org <1 %
Internet Source

40 www.myfoodresearch.com <1 %
Internet Source

41	Elham S. E. Saleh, Samar S. Tawfeek, Asmaa A. A. Abdel - Fadeel, Asmaa S. A. Abdel - Daim et al. " Effect of dietary protease supplementation on growth performance, water quality, blood parameters and intestinal morphology of Nile tilapia ( ) ", Journal of Animal Physiology and Animal Nutrition, 2021<br>Publication	<1 %
42	Hany M.R. Abdel-Latif, Mohamed M. Abdel-Daim, Mustafa Shukry, Joanna Nowosad, Dariusz Kucharczyk. "Benefits and applications of Moringa oleifera as a plant protein source in Aquafeed: A review", Aquaculture, 2022<br>Publication	<1 %
43	"Fishery Products", Wiley, 2009<br>Publication	<1 %
44	Ghasem Mohammadi, Gholamreza Rafiee, Mohammed F. El Basuini, Hien Van Doan et al. "Oregano (Origanum vulgare), St John's-wort (Hypericum perforatum), and lemon balm (Melissa officinalis) extracts improved the growth rate, antioxidative, and immunological responses in Nile tilapia (Oreochromis niloticus) infected with Aeromonas hydrophila", Aquaculture Reports, 2020<br>Publication	<1 %

45 Shi, Ze, Xiao-Qin Li, M.A. Kabir Chowdhury, Jia-Nan Chen, and Xiang-Jun Leng. "Effects of protease supplementation in low fish meal pelleted and extruded diets on growth, nutrient retention and digestibility of gibel carp, Carassius auratus gibelio", Aquaculture, 2016. <1%
Publication

Exclude quotes	On	Exclude matches	Off
Exclude bibliography	On		
